# State-of-the-art treatment alternatives for base of skull meningiomas: complementing and controversial indications for neurosurgery, stereotactic and robotic based radiosurgery or modern fractionated radiation techniques

**DOI:** 10.1186/1748-717X-7-226

**Published:** 2012-12-29

**Authors:** Stephanie E Combs, Ute Ganswindt, Robert L Foote, Douglas Kondziolka, Jörg-Christian Tonn

**Affiliations:** 1 Department of Radiation Oncology, University Hospital of Heidelberg, Im Neuenheimer Feld 400, 69120, Heidelberg, Germany; 2 Department of Radiation Oncology, Mayo Clinic, 200 First Street SW, Rochester, MN, MN 55905, USA; 3 Department of Neurosurgery, New York University, 530 First Avenue, 8R, New York, NY 10016, USA; 4 Department of Radiation Oncology, Ludwigs-Maximilians-University Munich, Marchioninistraße 15, Munich, 81377, Germany; 5 Department of Neurosurgery, Ludwigs-Maximilians-University Munich, Marchioninistraße 15, Munich, 81377, Germany

## Abstract

For skull base meningiomas, several treatment paradigms are available: Observation with serial imaging, surgical resection, stereotactic radiosurgery, radiation therapy or some combination of both. The choice depends on several factors. In this review we evaluate different treatment options, the outcome of modern irradiation techniques as well as the clinical results available, and establish recommendations for the treatment of patients with skull-base meningiomas.

## Introduction

There is controversy regarding the optimal treatment of patients with benign tumors of the skull base such as meningiomas. Different analyses focus on local control, morbidity and mortality of treatment, treatment duration and recovery times, as well as quality of life (QOL).

Skull base anatomy is intricate, and the close proximity of tumors to vital organs at risk (OAR) influence treatment decisions. Moreover, treatment recommendations depend on the experience of the surgeon or radiation oncologist, on available technology, and, last but not least, on the patients’ goals and preferences. In general, especially in the case of symptomatic lesions, treatment is mandatory for these usually benign tumors. However, in the case of an incidental finding or if clinical symptoms are mild and precise imaging characterization and definition can be obtained, observation may be chosen. This is particularly important in the elderly. In younger patients, observed patients must be followed closely because growth rates are variable. Studies show that annual growth rates are average, 1–3 mm per year. Such measurements do not reflect tumor volume changes and do not reflect changes in edema or continuing effects on brain tissue compression [1,2]. During the observation period, it is essential to keep patients in close follow-up, including contrast-enhanced imaging and clinical-neurological assessment including hearing evaluation, to minimize the risk of neglecting rapid growth, or development of severe symptoms. In general there is no urgent need for immediate surgical or radiotherapeutic intervention. However, when tumor growth can be documented, when clinical symptoms develop or worsen, or in younger patients who want to be treated at diagnosis, treatment may be indicated.

Various surgical approaches are available, depending on the size and location of the lesion, or on patient or surgeon specific factors. Radiation based strategies have become, in selected cases, a treatment alternative for meningiomas, especially in recurrent or progressive tumors with nonresectable remnants. This has improved with the development of precise treatment techniques such as radiosurgery (SRS) or fractionated stereotactic radiotherapy (FSRT), Intensity Modulated Radiotherapy (IMRT) or particle radiotherapy.

To date, however, no formal randomized study comparing surgical resection with radiotherapy techniques has been performed. Additionally, no study has prospectively assessed patient-related outcome by patient assessment and physician-based assessment, as well as quality of QOL after treatment.

Meningioma resection has long been considered the definitive treatment. Arguments for this approach include: Immediate removal of the lesion, rapid reduction of intracranial mass effect, and possibility to perform a precise pathological diagnosis. In general, radiation therapy was reserved for malignant tumors or for recurrences. Development and improvement in radiation therapy technology has altered this paradigm, and today, treatment decisions are more individualized, depending on the literature, physician’s preference and experience, as well as patient related factors. In particular, the large published experience using stereotactic radiosurgery since 1987 has altered how many think about meningiomas. When only resection or observation were available, many smaller tumors were observed, even after subtotal resection. This report provides a comprehensive review of the literature enhanced by center-specific experience in different treatment modalities leading to possible treatment algorithms for both indications.

## Surgical resection

Today, microsurgical techniques and a precise preoperative imaging allow surgical procedures to be highly effective with low procedure related morbidity [3]. Extensive, combined skull base approaches, although refined over the past two decades, are less frequently used nowadays since the paradigm of “preservation of function” has gained considerably more relevance [4,5]. Hence, the combination of more than one procedure using different approaches, each with low surgical morbidity, might be superior to one operation with disabling morbidity. Reconstruction of the sagittal sinus after “supramaximal” resection of falcine meningiomas involving this structure has been abandoned and replaced by irradiation of (progressing) remnants. Endoscopic techniques, either as a “stand alone” technique or as “endoscopic assisted microsurgery” have been introduced to subsequently lower the morbidity related to the surgical approach [6]. Altogether, the concept of functional preservation has led to a combination of surgical and radiotherapeutic techniques within the framework of a personalized therapy.

## Radiosurgery and fractionated radiation therapy

Over the years, irradiation techniques have evolved from conventional opposed field techniques, to 3D-conformal, CT-based radiotherapy to even more conformal treatments, such as fractionated stereotactic radiotherapy (FSRT) or single-fraction radiosurgery. The lattter techniques offer highly precise dose application to defined target volumes, with steep dose gradients to surrounding normal tissue. The main difference between one time treatment and fractionated regimens is based on biology: With radiosurgery, the dose is applied in a single fraction. Single session delivery has the potential for a greater tumoricidal response. Safety is based on the most precise radiation delivery using a stereotactic frame with judicious dose selection. Fractionated treatments exploit intrinsic repair mechanisms to reduce side effects to normal tissue. This holds especially true for larger volumes: For radiosurgery, a clear dose-volume-relationship has been shown, with risk for treatment-related side effects increasing with treatment volume [7]. On the other hand, a dose-dependent risk profile has been reported, and, depending on size and location of the lesion, generally marginal doses between 10–14 Gy are used [8-10]. Today, radiosurgery is administered using different techniques: Either dedicated devices such as the Gamma Knife™ (GK) or robotic-based radiosurgery using the Cyberknife-Technology™. Alternatively, stereotactically guided treatments using linear accelerator (LINAC)-based stereotactically based or frameless radiosurgery treatments are available. From the clinical perspective in the case of skull base meningiomas, all approaches have the potential to create comparable dose distributions with respect to dose conformity and sparing of normal tissue, therefore leading to comparable short-term clinical outcomes [11-13]. It is important to remember however that physicians do not all use these techniques similarly. Different forms of head/target fixation, different doses, different isodoses, different dose rates, different imaging-guidance techniques, and different approaches to critical regional anatomy lead to variability between centers.

Fractionated treatments are often applied as fractionated stereotactic radiotherapy, or modern high-precision image-guided radiotherapy (IGRT) approaches allowing for significantly improved precision over older radiotherapy paradigms. For complex shaped and larger lesions, the development of Intensity Modulated Radiotherapy (IMRT) offered another degree of freedom: As such, treatments consist of a multifield techniques with intensity modulated individual beams, improving dose conformality especially to complex shapes in very close vicinity to sensitive OAR.

**Figure 1:**
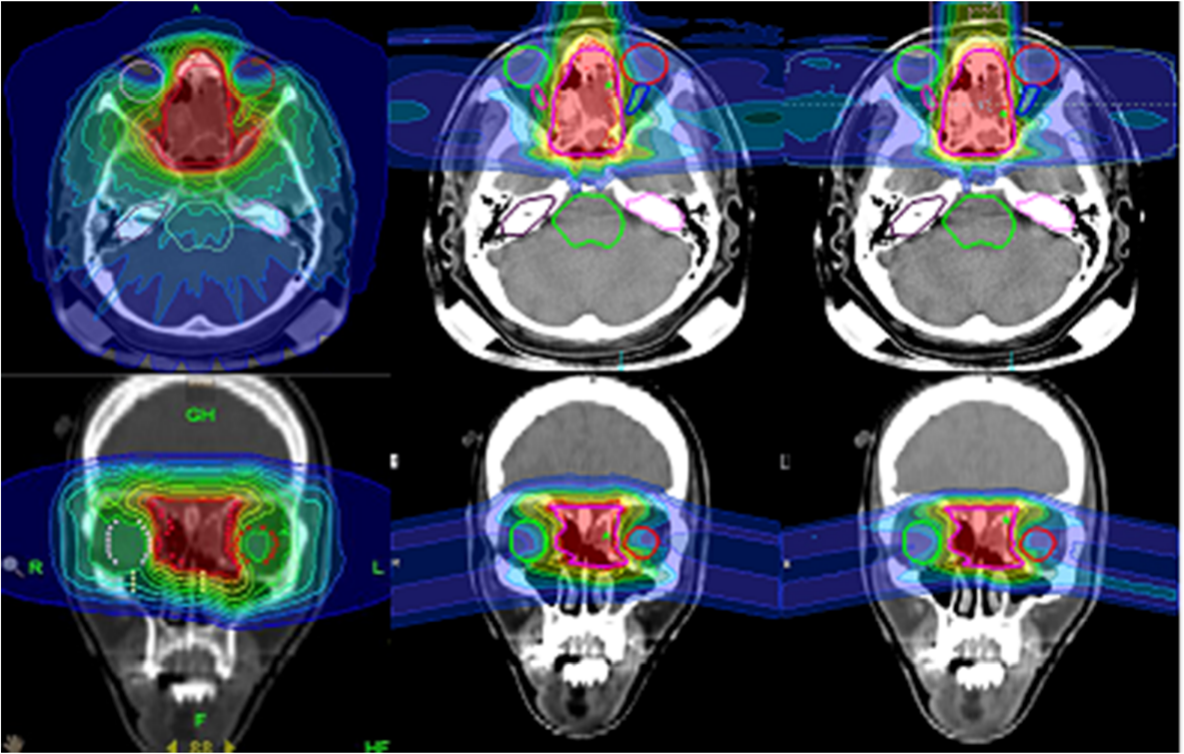
Treatment planning comparison of Helical Tomotherapy IMRT (left), scanned protons (middle) and scanned carbon ions (right).

Particle therapy is currently emerging since more and more treatment facilities are becoming clinically active. Its use in meningioma management is limited. With protons, reduction of integral dose to the patients can be achieved due to the physical properties of the beam, an inverted dose distribution with high local dose deposition within the Bragg Peak. The depth of this peak is energy dependent, leading to conformal dose distributions and reduction of entry and exit dose. However, proton based approaches have not typically been associated with the most conformal dose plans in clinical use. Again, this is user and center dependent. Like all elements of radiation techniques, this should be improved as treatment planning software works with device hardware. With higher-LET particles, such carbon ions, additionally biological properties can be exploited: Severe radiation damage to the cell nuclei contribute to an enhanced relative biological effectiveness (RBE). However, whether photons or protons are used, energy deposition is determined by dose. For certain indications, a clinical benefit of high-LET particles has been suggested, and for high-risk meningiomas the rational has been outlined and is currently being evaluated within a prospective clinical trial [14]. Modern facilities use active beam application, while older clinical data has been obtained using passive beam delivery. Besides biology, carbon ion beams are associated with less beam broadening along the beam channel, resulting in slightly improved dose conformality and sparing of OAR especially with deeper seated lesions. Figure 1 shows typical dose distributions for photon IMRT, protons and carbon ions for a patients with a skull base meningioma extending into the nasal cavity.

To conclusively report on clinical data of different radiation techniques, we performed a literature search in pubmed on the topic radiation therapy for the treatment of skull base meningiomas with the following key words:

(meningioma OR skull base meningiomas OR (meningiomas AND skull base)) AND (FSRT OR radiotherapy OR radiosurgery OR radiation OR IMRT OR proton) NOT case report NOT review. In total, 1237 citations were found.

We utilized the Cochrane Guidelines for the generation of review articles (http://www.Cochrane.org/resources/handbook) and chose only publications with a minimal patient number of 20 and a minimal follow-up time of 12 months to be able to better ascertain tumor control rates and late effects in addition to acute effects of treatment. Accordingly, case reports, reviews or technical reports were not included into this review.

Upon review, we excluded articles on optic nerve sheath meningiomas (ONSM), as well as cervical or non-skull-base intracranial meningiomas.

The techniques available in radiation oncology are reflected in 38 publications on FSRT, 49 publications on stereotactic radiosurgery, 68 publications on Gamma Knife or Cyberknife radiosurgery, and 3 reports on IMRT. For particle therapy, 10 articles could be found, predominantly on proton radiotherapy. Certain high-volume centers were identified for fractionated as well as radiosurgery techniques, from which several of the publications were derived. Two of the institutions with the most skull-base meningioma patients are represented as co-authors of the present review article.

### Radiosurgery

Independent of technique, radiosurgery approaches are comparable with respect to clinical outcome and toxicity. A clear volume-relationship for side effects has been reported, as well as a clear dose-dependency; this holds true for toxicity to cranial nerves, as well as for the development of intracranial edema [8,15,16]. Based on these data, limitations can be seen for complex volumes adjacent to OAR and with increasing size, while fractionated treatments are associated with a comparable dose profile independent of tumor volume or diameter [12,17-23]. This data obviously is based on the range of tumor volumes selected for treatment. On the other hand, for appropriate volumes, short term toxicity is very low; this, together with the short overall treatment times of radiosurgery, contributes to a high QOL as well as the possibility of patients returning to work quickly after treatment [24].

For radiosurgery, local control rates of 92-100% have been reported. Toxicity to the cranial nerves are commonly kept below 5%, depending on clinical factors. Even for meningiomas at the cerebello-pontine angle, facial nerve morbidity is rare. In the past, higher rates of side effects were observed, however, knowledge of normal-tissue tolerance and distinct tolerance differences between selected neural structures has been obtained. For example, due to the tolerance of the optic pathway, maximum point doses should be kept below 10 Gy (single fraction) to optic pathway structures. Early reports which advocated a <8 Gy approach, were likely too conservative. Older studies have shown that with 10–15 Gy, radiation induced optic neuropathy was around 30%, and approximately 80% with doses of 15 Gy or more [25], while doses of 12–16 Gy to only very small segments of the optic nerve were associated with acceptable toxicity [8]. This is supported by an extensive analysis by Mayo and colleagues, calculating a marked increase of risk at doses > 60 Gy at approximately 1.8 Gy/fraction and at >12 Gy for single-fraction radiosurgery [10]. Other neural structures, such as other cranial nerves, obviously tolerate slighty more dose [8]. Therefore, single dose prescription should aim at 12–18 Gy marginal coverage, however, individual increase or decrease in radiosurgical dose depends on tumor volume, tumor histology, tumor location, neighboring OAR, dose to OAR as well as individual pre-treatment factors such as pre-existing neurologgic deficits and or previous interventions.

The Pittsburgh group reported outcome in 168 patients with petroclival meningioma treated with GK radiosurgery [26]. Median dose to the tumor margin was 13 Gy; after a median follow-up of 72 months, neurological status improved in 44 patients (26%), remained stable in 98 (58%), and worsened in 26 (15%). Tumor volume decreased in 46% of the patients, and remained stable in 44%. Overall 5- and 10-year progression-free survival rates were 91 and 86%. Tumor volume = 8 cm^3^ was significantly associated with a higher risk of progression. Starke et al. supported these results, with local control rates of 98%, 96%, and 78% at 3, 5, and 10 years [27]. 51% of the patients showed decrease in tumor volume during follow-up, and in 36% volume stability was was observed. Failure was significantly associated with older age (> 65 years), and lower margin dose (mostly associated with larger treatment volumes). Of all, 91% of the patients demonstrated no change or improvement in their neurological condition.

A group of 251 patients were treated over a time span of 22 years with Gamma Knife radiosurgery [15]; of these, tumor size decreased in 181 patients (72.1%) and was unchanged in 67 patients (26.7%). The 3- and 10-year local control rate was 99.4%; only 3 patients (1.2%) had in-field tumor progression, and no marginal recurrences were observed. The 1- and 5-year complication rates were 8.3% and 11.5%, respectively. Radiation-related complications were associated with convexity/falx tumors, not in the skull base region, and increasing tumor volume on multivariate analysis.

Even for patients with brain stem compression or in critical locations such as the foramen magnum, radiosurgery can be considered as a treatment alternative on an individual decision basis, especially in older patients or patients with significant comorbidities [28,29]; in general, in these lesions, resection should be the first choice if feasible, since tumors are often associated with symptoms alleviated with surgery, and risk for side effects is considerably lower with more space between the tumor and brain stem. However, for selected cases, radiosurgery can be applied safely when marginal doses are kept below 15 Gy [9,30,31]. For example Nakaya et al. reported 246 patients with brainstem compression from benign skull-base tumors treated with Gamma Knife radiosurgery [29]. A median marginal dose of 13 Gy was prescribed. For meningiomas, median follow-up was 60 months. The tumor control rate was 100% for meningiomas. Symptoms improved in 43.2% of patients with meningioma.

Radiosurgery is a safe alternative for skull-base meningioma, independent of location, however, limitations must be kept in mind with close proximity to sensitive OAR as well as with increasing volumes. Thus, a preferential indications are smaller volumes.

### Fractionated stereotactic radiotherapy (FSRT)

Exploiting the radiobiological mechanisms of repair, fractionated treatment with 1.8 – 2 Gy per fraction offers the potential for a broader safety profile, especially for tumors involving sensitive OAR, directly adjacent to OAR, or with larger volumes. The benefit of fractionation is comparable for smaller and larger lesions, therefore, dose and volume margins are more variable due to a larger therapeutic window [32]. The special sensitivity of OAR, such as the optic nerves or chiasm, often lead to some compromise in dose prescription, potentially jeopardizing local control. In such locations, therefore, indications for radiosurgery versus fractionated treatment should be weighed against each other [33,34].

Several techniques are used, from stereotactic regimens, to image-guided highly precise approaches, with comparable outcomes. Early data by Maire et al. demonstrated neurological improvement in 72% patients with skull base meningiomas treated with FSRT; progression-free survival was 94%. Other studies with 24 to 72 patients report local control rates between 92-100%, with median doses of 50 to 56.7 Gy, and rates for permanent deficits of 1.6% to 9.8%, depending on series [19-21,35-40]. Most volumes were comparably large, between 14.5 and 57.2 ml. Minitti et al. could show that even for large volumes up to 150 ml, the 3-year and 5-year local control rates were 96% and 93% [22]. The Heidelberg group of 507 patients treated with either FSRT or IMRT reported local control of 91% at 10 years for benign meningiomas; QOL was unchanged in 47.7% of the patients, and 37.5% showed improvement. Most patients reported an improvement of symptoms or remain unchanged; in only a few patients disorders worsened over time or side effects developed [18].

After radiation therapy volume reduction can be observed over time: In 59 patients followed after FSRT, the mean size reduction was 17%, 23%, and 30% (at < 24 months, 24–48 months, and 48–72 months); the mean relative size reduction compared to the volume before radiotherapy was 27% [41]. Similar results were observed by Henzel et al., with a decrease in volume of 33% at 24 months and of 36% at 36 months after FSRT [20].

Assessment of neurocognitive functioning, which is of concern in radiation treatment of benign lesions, has been analyzed by independent groups: Steinvorth et al. performed a neuropsychological evaluation in 40 patients with skull base meningiomas [42]. Slight reduction in memory functioning during treatment was observed, followed by improvement in attention functions. No cognitive deterioration during follow-up was shown, and mood state improved after treatment. Nieuwenhuizen and co-workers revealed impaired neurocognitive functioning in meningioma patients after treatment, independent of surgery or other treatment modalities. However, they could show that additional radiation did not lead to a decline in neurocognitive functioning or QOL [43]. An analysis of self-reported outcome supported that most patients report stable or increasing QOL and improvement of symptoms during follow-up [18].

### Intensity Modulated Radiotherapy (IMRT)

Treatment planning comparisons have shown the benefits of IMRT compared to other 3D-treatments for several indications. For meningiomas, this holds true especially for larger volumes with complex anatomy [13]. In terms of Planning Target Volume (PTV) coverage, there is an advantage in using IMRT for all target shapes, but especially for irregularly shaped and and concave volumes. In some cases, IMRT can reduce dose to OAR, however, in total the volume of normal tissue receiving a low dose can be larger than with FSRT [44]. Assuming comparable target coverage and dose, short-term results after IMRT or FSRT are comparable [18]. A benefit in terms of dose distribution for IMRT is especially evident for highly complex anatomy, multiple lesions or meningiomatosis [17]. Outcome after IMRT-treated meningiomas is only available from a few institutions: Early data from Heidelberg had shown excellent local control of 94%, with improvement of preexisting neurological deficits in 40% of the patients [45,46]; stable disease was observed in 73.4%, and tumor volume reduction in 20.2% of the patients. Progression was evident in 6.4% during follow-up. Long-term follow-up of the same cohort of patients confirmed low rates of toxicity and high local control, with improved or unchanged QOL [18].

Thus, FSRT and IMRT provide excellent dose coverage, and choice of treatment should be made on an individual basis depending on the anatomy. It should be kept in mind that with FSRT integral dose might be lower than with IMRT, which is of importance for long-term outcomes especially in younger patients. Local control rates are high even for larger volume tumors, with low rates of side effects. Therefore, fractionated techniques offer excellent treatment options independent of size and location of the lesion.

### Particle therapy

Smaller series have been published on particle therapy, mostly protons, for skull base meningiomas [47-54]: All data compare well to photon series with FSRT, IMRT or radiosurgery, with local control ranging between 88-100% with low toxicity. As described above, protons are characterized by an inverted dose profile leading to a potential reduction of integral dose. Several studies and calculations have shown that this could lead to a significant reduction of long-term sequelae, such as neurocognitive dysfunctioning or secondary malignancies. Only recently, Arvold and colleagues compared proton with highly advanced photon plans and suggested a 50% reduction of the risk for radiation associated secondary malignancies; they also suggested a, significant dose reduction to neurocognitive and other critical structures [55]. This theoretical model remains to be evaluated. On the other hand, no significant difference in anticipated late effects as calculated by NTCP models was determined, which was below 1% for all modalities. To date, however, no controlled clinical trial has been performed to confirm a benefit for skull base tumors, and considering the required endpoints and long-term horizon of follow-up this might be difficult to determine. When evaluating the dose distributions, perhaps such trials are not required, especially when considering that future proton treatment centers and devices are potentially decreasing in cost and in the long-run resembling modern photon radiotherapy in terms of cost. However, to date, differences in cost and availibility drive such discussions and critical evaluation of data is necessary. It is important to recall that a benign meningioma presents a situation, unlike glioma or metastases, where all tissue outside the imaging-defined target, is normal. Thus, with any technique, an optimum goal is to deliver as little radiation as possible to any structure outside the tumor. For smaller volumes this is more readily achieved. With radiosurgery, the most conformal and selective dose plans with rigid target fixation, achieve this. With all approaches, this goal is crucial. Although individual centers using different techniques can advocate the value of the approach they utilize from a physics perspective, only long-term clinical outcomes of safety and efficacy are truly important.

### Treatment planning for radiation therapy

Independent of technique and modality, exact target volume definition is an essential prerequisite for modern highly precise and selective radiotherapy. For low-grade meningiomas, it has been shown that location of local recurrences is mainly outfield, i.e. commonly regions receiving less than 90% of the prescribed dose [56]. These results support established dose concepts as described above, and stress the necessity of reliable imaging-based planning. For radiosurgery, smaller volume definition is commonly performed, not including much of the dural tail. With fractionated techniques, generally, regions of the dural tail are included into the GTV, and therefore, should be identified as meningioma and distinguished from other reactive changes of the dura. Interestingly, recurrence from the “dural tail” is uncommon. Biopsies of dural tail tissue at resection tend to show hyperemic dura and not tumor.

Meningiomas are best seen on MRI with contrast. For selected basal tumors, fat suppression imaging is helpful. CT or PET can be adjuncts in selected cases, For bony structures, i.e. infiltration of cavernous sinus or other regions of the skull base, CT imaging can best show infiltration and osteolytic areas of bony infiltration. Additional information is provided by contrast-enhanced MRI, especially on T1-weighted imaging, best for delineation of macroscopically visible meningiomas. Combined or fused MR-CT may assist in tumor delineation especially when the bony base of the skull obscures the target in CT images [57].

Especially in regions of infiltration into musculature such as pterygoid region, of along dural pathways, it is often difficult to define exact borders. Additional imaging such as amino-acid-PET or somatostatin-receptor-PET may help distinguish tumor from inflammation and edema.

**Figure 2:**
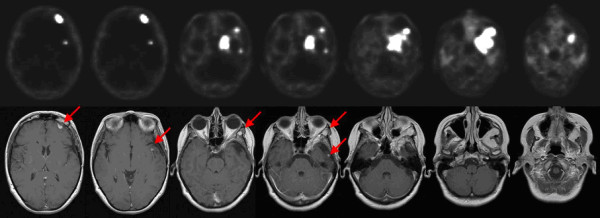
**Multilocular relapse of a skull base meningioma 9 years after surgery in a 46-year old woman.** 68-Ga-DOTATOC-PET (above) and T1 weighted MRI (below). Red arrows show distant lesions initially detected by PET imaging.

A study on [11]C-methionine positron emission tomography (MET-PET) for gross tumor volume delineation in fractionated stereotactic radiotherapy of skull base meningiomas demonstrated that addition of MET-PET can lead to an increase, as well as a decrease in Gross Target Volume (GTV) [58]. Additionally, the same group could show that inter-observer variability or target volume definition was reduced significantly when adding MET-PET to CT-MRI-based contouring; with additional PET-information the target volumes were almost identical, while prominent differences in volumes was seen without PET-information [59]. Besides amino acid PET, [(68)Ga]-DOTA-D Phe[1]-Tyr[3]-Octreotide (DOTATOC)-PET can be used for treatment planning of meningiomas (Figure 2). Within a group of 26 patients receiving 68-Ga-DOTATOC-PET/CT for radiation treatment planning additional information on tumor extension was seen in 17/26 patients; moreover, median GTV was significantly larger after integration of PET, on case-by-case basis however enlargements, as well as reduction of GTV and CTV volumes were seen [60]. Most adaptations were seen in the postoperative setting, or in regions of bony anatomy. Graf et al. confirmed the influence of 68-Ga-DOTATOC-PET in 54 lesions, mostly showing an increase in GTV with PET-imaging [61]. In the Heidelberg and Munich center, 68-Ga-DOTATOC-PET is standard-of-care for treatment planning of meningiomas based on the initial implementation of the tracer in this patient population. Besides volume optimization, 68-Ga-DOTATOC-PET can help in detection of meningiomas, especially smaller lesions or lesions in direct vicinity of bony structures.

## Timing of resection or irradiation

Based on the clinical data provided above, the indication both for surgical resection as well as radiation therapy or radiosurgery is based on individual factors, such as patient age and comorbidities, the presence of clinical symptoms, volume and affected anatomical areas, mass effect of the lesion or compression of OAR, known growth rate, as well as the patient’s preference.

Patients with aymptomatic lesions with typical imaging of a benign skull base meningioma can be offered a wait-and-scan program including thorough regular clinical-neurological and opthalmological assessment, as well as contrast-enhanced MR-imaging. However if a tumor is optimal for irradiation now, and subsequent growth would eliminate this option, then irradiation may be a good choice now for that patient. For typical cranial base meningiomas, low kinetics of growth or expansion have been observed, and they can appear as indolent lesions over long time spans [2]. Some, however, grow more rapidly. On the other hand, if the lesion is resectable, a neurosurgical intervention can be performed, if the patient prefers immediate treatment. Early radiation therapy can be discussed, however, in asymptomatic patients the minimal residual risk for side effects should be weighed against arguments for early treatment. In patients with clinical symptoms or observed significant growth, therapy should be recommended:

In some lesions, both resection and radiosurgery can be offered as equivalent treatment options in appropriately selected patients, depending on the patient’s preference and the technical availabilities. This holds especially true for those lesions extending into critical regions such as the cavernous sinus, where a complete resection would only be possible with high rates of treatment-related side effects. Here, radiotherapy/radiosurgery of the tumor within the cavernous sinus is superior in terms of benefit/risk ratio. While surgical resection offers the benefit of subsequent pathological evaluation of the specimen, excluding the remaining risk of 1-2% considered as “diagnostic miss” with imaging-based diagnosis. However, with multimodal imaging including CT, MRI and PET this risk remains to be below 5%, and with DOTATOC-PET imaging which is highly specific the risk is negligible.

For symptomatic patients with mass effect or OAR compression, surgery should be performed; interdisciplinary discussion might decide on planned partial resections and subsequent wait-and-see or postoperative radiation therapy to reduce mass effect but to reduce treatment-related toxicity, such as with cavernous sinus infiltration.

Controversy exists on early postoperative radiation therapy versus a wait-and-see strategy postoperatively. Some data can be used to argue for early treatment to improve and prolong local control when compared to surgery alone, supporting the idea that prolongation of local control can be achieved especially after partial resection [35,62]. However, radiation performed as salvage-treatment after surgery is comparably effective, and might be withheld to minimize side effects and spare patients from additional treatment that might not be necessary [18]. Ideally, at surgery one hopes to resect the *entire* tumor. Alternatively, combined resection followed by irradiation of any remnant, provides a planned approach for the *entire* tumor, using mechanical and biologic means.

In this regard histological evidence of atypical or anaplastic meningioma (WHO II/III, =10% of all meningiomas) offers different challenges. Knowledge of high-grade biology changes the treatment paradigm compared to benign lesions. This subgroup has tended to be more aggressive and has been associated with a much poorer outcome [63,64]. Thus, postoperative radiotherapy is rather recommended for these patients. In contrast to minimized stereotactic target volume approaches in benign meningiomas the PTV for atypical and anaplastic meningiomas is enlarged to the resection cavity plus a safety margin of approx. 1–2 cm. Additionally, the benefit of postoperative radiotherapy in this subgroup is related to greater radiation doses of 60–66 Gy [65-68]. Interestingly, higher grade meningiomas are rare at the skull base and are much more common in convexity or parasagittal tumors.

**Figure 3:**
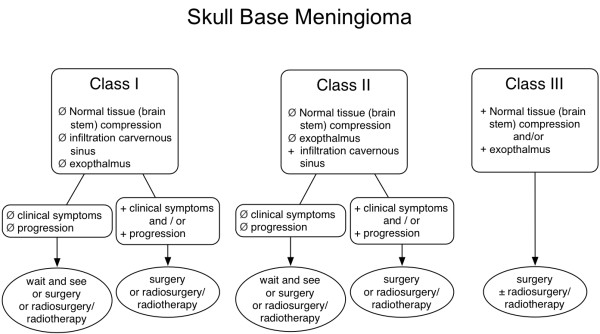
**Treatment algorithm for skull base meningiomas modified from Combs SE et al. Radiother Oncol 2012[**[18]**].**

In summary, several factors influence decision making in benign-skull base meningiomas. The treatment algorithm in Figure 3 outlines recommendations for councelling patients and on selection of a possible treatment regimen.

## Competing interests

The authors declare that they have no competing interests.

## Authors’ contributions

SEC made the manuscript concept, performed the literature search and drafted the review on radiation oncology; JCT drafted the neurosurgery section of the manuscript; UG, RLF and DK added important content and included data from their institution. All authors read and approved the final version of the manuscript.

## References

[B1] Arthurs BJ Fairbanks RK Demakas JJ Lamoreaux WT Giddings NA Mackay AR A review of treatment modalities for vestibular schwannoma Neurosurg Rev 2011 34 265 277 10.1007/s10143-011-0307-8 21305333

[B2] Bindal R Goodman JM Kawasaki A Purvin V Kuzma B The natural history of untreated skull base meningiomas Surg Neurol 2003 59 87 92 10.1016/S0090-3019(02)00995-3 12648902

[B3] Rachinger W Grau S Tonn JC Different microsurgical approaches to meningiomas of the anterior cranial base Acta Neurochir (Wien) 2010 152 931 939 10.1007/s00701-010-0646-1 20383724

[B4] Westphal M Lamszus K Tonn JC Tonn JC, Westphal M, Rutka J Meningiomas and meningeal tumors Oncology of CNS tumors 2010 2 Heidelberg, New York: Springer 95 118

[B5] Pechlivanis I Wawrzyniak S Engelhardt M Schmieder K Evidence level in the treatment of meningioma with focus on the comparison between surgery versus radiotherapy. A review J Neurosurg Sci 2011 55 319 328 22198584

[B6] Komotar RJ Starke RM Raper DM Anand VK Schwartz TH Endoscopic endonasal versus open transcranial resection of anterior midline skull base meningiomas World Neurosurg 2012 77 713 724 10.1016/j.wneu.2011.08.025 22120296

[B7] Kang CS Zheng LG Xu DS Dose-volume effect in gamma knife radiosurgery of meningiomas Stereotact Funct Neurosurg 1999 73 72 78 10.1159/000029758 10853105

[B8] Morita A Coffey RJ Foote RL Schiff D Gorman D Risk of injury to cranial nerves after gamma knife radiosurgery for skull base meningiomas: experience in 88 patients J Neurosurg 1999 90 42 49 10.3171/jns.1999.90.1.0042 10413154

[B9] Flickinger JC Kondziolka D Niranjan A Lunsford LD Dose selection in stereotactic radiosurgery Prog Neurol Surg 2007 20 28 42 17317974 10.1159/000100093

[B10] Mayo C Martel MK Marks LB Flickinger J Nam J Kirkpatrick J Radiation dose-volume effects of optic nerves and chiasm Int J Radiat Oncol Biol Phys 2010 76 S28 S35 10.1016/j.ijrobp.2009.07.1753 20171514

[B11] Theelen A Martens J Bosmans G Houben R Jager JJ Rutten I Relocatable fixation systems in intracranial stereotactic radiotherapy. Accuracy of serial CT scans and patient acceptance in a randomized design Strahlenther Onkol 2012 188 84 90 10.1007/s00066-011-0018-7 22194025

[B12] Debus J Wuendrich M Pirzkall A Hoess A Schlegel W Zuna I High efficacy of fractionated stereotactic radiotherapy of large base-of-skull meningiomas: long-term results J Clin Oncol 2001 19 3547 3553 11481362 10.1200/JCO.2001.19.15.3547

[B13] Pirzkall A Carol M Lohr F Hoss A Wannenmacher M Debus J Comparison of intensity-modulated radiotherapy with conventional conformal radiotherapy for complex-shaped tumors Int J Radiat Oncol Biol Phys 2000 48 1371 1380 10.1016/S0360-3016(00)00772-0 11121636

[B14] Combs SE Edler L Burkholder I Rieken S Habermehl D Jakel O Treatment of patients with atypical meningiomas Simpson grade 4 and 5 with a carbon ion boost in combination with postoperative photon radiotherapy: the MARCIE trial BMC Cancer 2010 10 615 10.1186/1471-2407-10-615 21062428 PMC2996393

[B15] Pollock BE Stafford SL Link MJ Garces YI Foote RL Single-fraction radiosurgery for presumed intracranial meningiomas: efficacy and complications from a 22-year experience Int J Radiat Oncol Biol Phys 2012 83 1414 1418 10.1016/j.ijrobp.2011.10.033 22209154

[B16] Novotny J Jr Kollova A Liscak R Prediction of intracranial edema after radiosurgery of meningiomas J Neurosurg 2006 105 Suppl 120 126 18503344 10.3171/sup.2006.105.7.120

[B17] Combs SE Sterzing F Uhl M Habl G Schubert K Debus J Helical tomotherapy for meningiomas of the skull base and in paraspinal regions with complex anatomy and/or multiple lesions Tumori 2011 97 484 491 21989438 10.1177/030089161109700412

[B18] Combs SE Adeberg S Dittmar JO Welzel T Rieken S Habermehl D Skull base meningiomas: Long-term results and patient self-reported outcome in 507 patients treated with fractionated stereotactic radiotherapy (FSRT) or intensity modulated radiotherapy (IMRT) Radiother Oncol 2012 Aug. 17; epub ahead of print 10.1016/j.radonc.2012.07.008 22906549

[B19] Hamm K Henzel M Gross MW Surber G Kleinert G Engenhart-Cabillic R Radiosurgery/stereotactic radiotherapy in the therapeutical concept for skull base meningiomas Zentralbl Neurochir 2008 69 14 21 10.1055/s-2007-992138 18393160

[B20] Henzel M Gross MW Hamm K Surber G Kleinert G Failing T Significant tumor volume reduction of meningiomas after stereotactic radiotherapy: results of a prospective multicenter study Neurosurgery 2006 59 1188 1194 17277681 10.1227/01.NEU.0000245626.93215.F6

[B21] Henzel M Gross MW Hamm K Surber G Kleinert G Failing T Stereotactic radiotherapy of meningiomas: symptomatology, acute and late toxicity Strahlenther Onkol 2006 182 382 388 10.1007/s00066-006-1535-7 16826356

[B22] Minniti G Clarke E Cavallo L Osti MF Esposito V Cantore G Fractionated stereotactic conformal radiotherapy for large benign skull base meningiomas Radiat Oncol 2011 6 36 10.1186/1748-717X-6-36 21486436 PMC3094366

[B23] Minniti G Amichetti M Enrici RM Radiotherapy and radiosurgery for benign skull base meningiomas Radiat Oncol 2009 4 42 10.1186/1748-717X-4-42 19828022 PMC2768735

[B24] Chao ST Thakkar VV Barnett GH Vogelbaum MA Angelov L Weil RJ Prospective study of the short-term adverse effects of gamma knife radiosurgery Technol Cancer Res Treat 2012 11 117 122 22335405 10.7785/tcrt.2012.500240

[B25] Leber KA Bergloff J Pendl G Dose–response tolerance of the visual pathways and cranial nerves of the cavernous sinus to stereotactic radiosurgery J Neurosurg 1998 88 43 50 10.3171/jns.1998.88.1.0043 9420071

[B26] Flannery TJ Kano H Lunsford LD Sirin S Tormenti M Niranjan A Long-term control of petroclival meningiomas through radiosurgery J Neurosurg 2010 112 957 964 10.3171/2009.8.JNS09695 19731986

[B27] Starke RM Nguyen JH Rainey J Williams BJ Sherman JH Savage J Gamma Knife surgery of meningiomas located in the posterior fossa: factors predictive of outcome and remission J Neurosurg 2011 114 1399 1409 21214335 10.3171/2010.11.JNS101193

[B28] Starke RM Nguyen JH Reames DL Rainey J Sheehan JP Gamma knife radiosurgery of meningiomas involving the foramen magnum J Craniovertebr Junction Spine 2010 1 23 28 10.4103/0974-8237.65478 20890411 PMC2944857

[B29] Nakaya K Niranjan A Kondziolka D Kano H Khan AA Nettel B Gamma knife radiosurgery for benign tumors with symptoms from brainstem compression Int J Radiat Oncol Biol Phys 2010 77 988 995 10.1016/j.ijrobp.2009.06.089 20381265

[B30] Kondziolka D Flickinger JC Lunsford LD The principles of skull base radiosurgery Neurosurg Focus 2008 24 E11 18447740 10.3171/FOC/2008/24/5/E11

[B31] Kondziolka D Mathieu D Lunsford LD Martin JJ Madhok R Niranjan A Radiosurgery as definitive management of intracranial meningiomas Neurosurgery 2008 62 53 58 10.1227/01.NEU.0000311061.72626.0D 18300891

[B32] Ernst-Stecken A Lambrecht U Ganslandt O Mueller R Fahlbusch R Sauer R Radiosurgery of small skull-base lesions. No advantage for intensity-modulated stereotactic radiosurgery versus conformal arc technique Strahlenther Onkol 2005 181 336 344 10.1007/s00066-005-1371-1 15900431

[B33] Shrieve DC Hazard L Boucher K Jensen RL Dose fractionation in stereotactic radiotherapy for parasellar meningiomas: radiobiological considerations of efficacy and optic nerve tolerance J Neurosurg 2004 101 Suppl 3 390 395 15537194

[B34] Sibtain A Plowman PN Stereotactic radiosurgery. VII. Radiosurgery versus conventionally-fractionated radiotherapy in the treatment of cavernous sinus meningiomas Br J Neurosurg 1999 13 158 166 10.1080/02688699943925 10616585

[B35] Compter I Zaugg K Houben RM Dings JT Bosmans G Buescher C High symptom improvement and local tumor control using stereotactic radiotherapy when given early after diagnosis of meningioma. A multicentre study Strahlenther Onkol 2012 188 887 893 10.1007/s00066-012-0155-7 22961046

[B36] Alheit H Saran FH Warrington AP Rosenberg I Perks J Jalali R Stereotactically guided conformal radiotherapy for meningiomas Radiother Oncol 1999 50 145 150 10.1016/S0167-8140(98)00133-9 10368037

[B37] Maguire PD Clough R Friedman AH Halperin EC Fractionated external-beam radiation therapy for meningiomas of the cavernous sinus Int J Radiat Oncol Biol Phys 1999 44 75 79 10.1016/S0360-3016(98)00558-6 10219797

[B38] Jalali R Loughrey C Baumert B Perks J Warrington AP Traish D High precision focused irradiation in the form of fractionated stereotactic conformal radiotherapy (SCRT) for benign meningiomas predominantly in the skull base location Clin Oncol (R Coll Radiol) 2002 14 103 109 10.1053/clon.2001.0040 12069116

[B39] Selch MT Ahn E Laskari A Lee SP Agazaryan N Solberg TD Stereotactic radiotherapy for treatment of cavernous sinus meningiomas Int J Radiat Oncol Biol Phys 2004 59 101 111 10.1016/j.ijrobp.2003.09.003 15093905

[B40] Onodera S Aoyama H Katoh N Taguchi H Yasuda K Yoshida D Long-term outcomes of fractionated stereotactic radiotherapy for intracranial skull base benign meningiomas in single institution Jpn J Clin Oncol 2011 41 462 468 10.1093/jjco/hyq231 21177777

[B41] Astner ST Theodorou M Dobrei-Ciuchendea M Auer F Kopp C Molls M Tumor shrinkage assessed by volumetric MRI in the long-term follow-up after stereotactic radiotherapy of meningiomas Strahlenther Onkol 2010 186 423 429 10.1007/s00066-010-2138-x 20803282

[B42] Steinvorth S Welzel G Fuss M Debus J Wildermuth S Wannenmacher M Neuropsychological outcome after fractionated stereotactic radiotherapy (FSRT) for base of skull meningiomas: a prospective 1-year follow-up Radiother Oncol 2003 69 177 182 10.1016/S0167-8140(03)00204-4 14643955

[B43] van Nieuwenhuizen D Klein M Stalpers LJ Leenstra S Heimans JJ Reijneveld JC Differential effect of surgery and radiotherapy on neurocognitive functioning and health-related quality of life in WHO grade I meningioma patients J Neurooncol 2007 84 271 278 10.1007/s11060-007-9366-7 17431545

[B44] Baumert BG Norton IA Davis JB Intensity-modulated stereotactic radiotherapy vs. stereotactic conformal radiotherapy for the treatment of meningioma located predominantly in the skull base Int J Radiat Oncol Biol Phys 2003 57 580 592 10.1016/S0360-3016(03)00587-X 12957272

[B45] Pirzkall A Debus J Haering P Rhein B Grosser KH Hoss A Intensity modulated radiotherapy (IMRT) for recurrent, residual, or untreated skull-base meningiomas: preliminary clinical experience Int J Radiat Oncol Biol Phys 2003 55 362 372 10.1016/S0360-3016(02)03809-9 12527049

[B46] Milker-Zabel S Zabel-du BA Huber P Schlegel W Debus J Intensity-modulated radiotherapy for complex-shaped meningioma of the skull base: long-term experience of a single institution Int J Radiat Oncol Biol Phys 2007 68 858 863 10.1016/j.ijrobp.2006.12.073 17379447

[B47] Boskos C Feuvret L Noel G Habrand JL Pommier P Alapetite C Combined proton and photon conformal radiotherapy for intracranial atypical and malignant meningioma Int J Radiat Oncol Biol Phys 2009 75 399 406 10.1016/j.ijrobp.2008.10.053 19203844

[B48] Gudjonsson O Blomquist E Nyberg G Pellettieri L Montelius A Grusell E Stereotactic irradiation of skull base meningiomas with high energy protons Acta Neurochir (Wien) 1999 141 933 940 10.1007/s007010050399 10526074

[B49] Halasz LM Bussiere MR Dennis ER Niemierko A Chapman PH Loeffler JS Proton stereotactic radiosurgery for the treatment of benign meningiomas Int J Radiat Oncol Biol Phys 2011 81 1428 1435 10.1016/j.ijrobp.2010.07.1991 20934263

[B50] Noel G Bollet MA Calugaru V Feuvret L Haie-Meder C Dhermain F Functional outcome of patients with benign meningioma treated by 3D conformal irradiation with a combination of photons and protons Int J Radiat Oncol Biol Phys 2005 62 1412 1422 10.1016/j.ijrobp.2004.12.048 16029801

[B51] Vernimmen FJ Harris JK Wilson JA Melvill R Smit BJ Slabbert JP Stereotactic proton beam therapy of skull base meningiomas Int J Radiat Oncol Biol Phys 2001 49 99 105 10.1016/S0360-3016(00)01457-7 11163502

[B52] Weber DC Lomax AJ Rutz HP Stadelmann O Egger E Timmermann B Spot-scanning proton radiation therapy for recurrent, residual or untreated intracranial meningiomas Radiother Oncol 2004 71 251 258 10.1016/j.radonc.2004.02.011 15172139

[B53] Weber DC Schneider R Goitein G Koch T Ares C Geismar JH Spot Scanning-based Proton Therapy for Intracranial Meningioma: Long-term Results from the Paul Scherrer Institute Int J Radiat Oncol Biol Phys 2011 10.1016/j.ijrobp.2011.08.027 22138457

[B54] Wenkel E Thornton AF Finkelstein D Adams J Lyons S De La MS Benign meningioma: partially resected, biopsied, and recurrent intracranial tumors treated with combined proton and photon radiotherapy Int J Radiat Oncol Biol Phys 2000 48 1363 1370 10.1016/S0360-3016(00)01411-5 11121635

[B55] Arvold ND Niemierko A Broussard GP Adams J Fullerton B Loeffler JS Projected second tumor risk and dose to neurocognitive structures after proton versus photon radiotherapy for benign meningioma Int J Radiat Oncol Biol Phys 2012 83 e495 e500 10.1016/j.ijrobp.2011.10.056 22285662

[B56] Askoxylakis V Zabel-du BA Schlegel W Debus J Huber P Milker-Zabel S Patterns of failure after stereotactic radiotherapy of intracranial meningioma J Neurooncol 2010 98 367 372 10.1007/s11060-009-0084-1 20012910

[B57] Schad LR Gademann G Knopp M Zabel HJ Schlegel W Lorenz WJ Radiotherapy treatment planning of basal meningiomas: improved tumor localization by correlation of CT and MR imaging data Radiother Oncol 1992 25 56 62 10.1016/0167-8140(92)90196-2 1410591

[B58] Astner ST Dobrei-Ciuchendea M Essler M Bundschuh RA Sai H Schwaiger M Effect of 11C-methionine-positron emission tomography on gross tumor volume delineation in stereotactic radiotherapy of skull base meningiomas Int J Radiat Oncol Biol Phys 2008 72 1161 1167 10.1016/j.ijrobp.2008.02.058 18440729

[B59] Grosu AL Weber WA Astner ST Adam M Krause BJ Schwaiger M 11C-methionine PET improves the target volume delineation of meningiomas treated with stereotactic fractionated radiotherapy Int J Radiat Oncol Biol Phys 2006 66 339 344 10.1016/j.ijrobp.2006.02.047 16765533

[B60] Gehler B Paulsen F Oksuz MO Hauser TK Eschmann SM Bares R [68 Ga]-DOTATOC-PET/CT for meningioma IMRT treatment planning Radiat Oncol 2009 4 56 10.1186/1748-717X-4-56 19922642 PMC2785827

[B61] Graf R Nyuyki F Steffen IG Michel R Fahdt D Wust P Contribution of (68)Ga-DOTATOC PET/CT to Target Volume Delineation of Skull Base Meningiomas Treated With Stereotactic Radiation Therapy 2012 Phys: Int J Radiat Oncol Biol 10.1016/j.ijrobp.2012.03.021 22575489

[B62] DeMonte F Smith HK al Mefty O Outcome of aggressive removal of cavernous sinus meningiomas J Neurosurg 1994 81 245 251 10.3171/jns.1994.81.2.0245 8027808

[B63] Maier H Ofner D Hittmair A Kitz K Budka H Classic, atypical, and anaplastic meningioma: three histopathological subtypes of clinical relevance J Neurosurg 1992 77 616 623 10.3171/jns.1992.77.4.0616 1527622

[B64] Salcman M Al-Mefty O Malignant meningiomas Meningiomas 75 New York: Raven 85 1991 Ref Type: Abstract

[B65] Goyal LK Suh JH Mohan DS Prayson RA Lee J Barnett GH Local control and overall survival in atypical meningioma: a retrospective study Int J Radiat Oncol Biol Phys 2000 46 57 61 10.1016/S0360-3016(99)00349-1 10656373

[B66] Aghi MK Carter BS Cosgrove GR Ojemann RG Amin-Hanjani S Martuza RL Long-term recurrence rates of atypical meningiomas after gross total resection with or without postoperative adjuvant radiation Neurosurgery 2009 64 56 60 10.1227/01.NEU.0000330399.55586.63 19145156

[B67] Dziuk TW Woo S Butler EB Thornby J Grossman R Dennis WS Malignant meningioma: an indication for initial aggressive surgery and adjuvant radiotherapy J Neurooncol 1998 37 177 188 10.1023/A:1005853720926 9524097

[B68] Engenhart-Cabillic R Farhoud A Sure U Heinze S Henzel M Mennel HD Clinicopathologic features of aggressive meningioma emphasizing the role of radiotherapy in treatment Strahlenther Onkol 2006 182 641 646 10.1007/s00066-006-1555-3 17072521

